# Impact of Tissue Enolase 1 Protein Overexpression in Esophageal Cancer Progression

**DOI:** 10.7150/ijms.52688

**Published:** 2021-01-26

**Authors:** Anh Tuan Hoang, Barbara Vizio, Luigi Chiusa, Antonio Cimino, Dino Solerio, Nhu Hon Do, Stefano Pileci, Michele Camandona, Graziella Bellone

**Affiliations:** 1 Department of Medical Sciences, University of Turin, 10126 Turin, Italy.; 2 Pathology Unit, AOU City of Health and Science of Turin, 10126 Turin, Italy.; 3 Department of Surgical Sciences, University of Turin, Unit of Digestive and Oncological Surgery 1U, AOU City of Health and Science of Turin, 10126 Turin, Italy.; 4 Vietnam National Institute of Ophthalmology, Hanoi, Vietnam.

**Keywords:** Enolase 1, esophageal cancer, Barrett's esophagus, tumor progression.

## Abstract

Enolase (ENO) 1 is a key glycolytic enzyme and important player in tumorigenesis. ENO1 overexpression has been correlated with tumor progression and/or worse prognosis in several solid malignancies. However, data concerning the impact of ENO1 in cancer conflict. The study correlated local and circulating ENO1 protein levels in esophageal cancer (EC) with clinicopathological data, to assess its potential clinical value. ENO1 expression was analyzed by immunohistochemistry in paired tumor and non-tumor tissue samples from 40 EC cases and mucosal biopsies from 45 Barrett's esophagus (BE) cases, plus in plasma from these patients and 25 matched healthy controls. ENO1 was abnormally elevated in cancer-cell cytoplasm in both EC types, in esophageal squamous cell carcinoma and in adenocarcinoma (EAC), increasing significantly with tumor stage progression and the transition from BE to EAC. EAC patients exhibited significantly lower ENO1 plasma concentrations than normal subjects. Neither local nor systemic ENO1 expression levels were significantly associated with overall survival. These results indicate ENO1 as potential biomarker, delineating a population of patients with Barrett's esophagus at high risk of cancer, and as new therapeutic opportunity in EC patient management. However, further confirmation might be necessary.

## Introduction

Esophageal cancer (EC) ranks eighth among common cancers and sixth among all cancer-related mortality worldwide 1. Overall survival is below 10% and, despite recent advances in therapeutic strategies, the 5-year survival rate of patients who have undergone resection is 20%-40% 2. Neither systemic neoadjuvant (chemotherapy before "curative" surgery) nor adjuvant therapy (chemotherapy after "curative" surgery) have shown a substantial effect on survival 3. Local control of EC can rarely be achieved, due to early micrometastatic spread, despite systemically-acting drugs 4.

The most common type of EC is esophageal squamous cell carcinoma (ESCC), with increasing morbidity in Western countries 5. The other type is esophageal adenocarcinoma (EAC), which arises in the setting of Barrett's esophagus (BE), characterized by the replacement of healthy esophageal epithelium with metaplastic columnar cells 6. The two subtypes are distinct entities, with some overlap concerning epidemiologic distribution, risk factors, pathogenesis, early tumor cell dissemination and metastasis, resulting in equally poor survival 7.

Enolase (ENO), also known as phosphopyruvate hydratase, was originally characterized as an enzyme involved in glycolytic metabolism 8. Recent findings have shown that ENO1 plays an important role in several biological and pathophysiological processes 9. In particular, upregulation of ENO1 has been reported in many different types of cancer 10-18, and is considered to be a key protein in tumorigenesis, cancer cell invasion, and metastasis 19, 20.

The expression levels of ENO1 in patients with EC have not yet been investigated. The study aimed to investigate the differential ENO1 protein expression in tumor and in adjacent non-cancerous tissue specimens, as well as in plasma from a series of primary EC, in BE patients and in matched healthy controls, in order to explore its clinicopathological relevance. The findings show that ENO1 is not a predictor of overall survival, but that it is a predictor of tumor progression, suggesting that it may help to select patients with more aggressive disease who may benefit from targeted therapies.

## Materials and Methods

### EC patients

Forty patients with EC, who had undergone esophagectomy at the Department of Surgical Sciences, University of Turin, Digestive and Oncological Surgery Unit 1U, AOU City of Health and Science of Turin, between January 2011 and June 2017, were qualified and included for our retrospective study approved by Institutional Ethics Committee of AOU City of Health and Science of Turin. None underwent anticancer treatment before entering the study, which was conducted under strict observance of the principles of the Declaration of Helsinki. Data including age, sex, tumor location and size, treatment protocol, curability, histology, tumor node metastasis (TNM), stage, and outcome were obtained from clinical and pathologic records. Surgical resections, clinical staging, and histopathologic classification were defined following the UICC-TNM system 21. Final pathologic staging was determined for all patients. Table 1 gives clinicopathologic characteristics of the patients. Duration of follow-up was from time of surgery to death, dropout, or end of December 2019. During the follow-up period, 9 (22.5%) tumors relapsed; 30 patients (75%) died; 28 of these (93.3%) died of the disease and 2 (6.7%) of other causes.

### BE patients

Forty-five patients with BE, who underwent upper endoscopy and biopsies as part of routine dysplasia surveillance or evaluation of upper gastrointestinal symptoms at the Endoscopy and Intestinal Motility Service, University of Turin, Italy, were qualified and included for this study, conducted under strict observance of the principles of the Declaration of Helsinki. All patients had originally been referred for endoscopic evaluation because of one or more of the following symptoms: dysphagia, heartburn, noncardiac chest pain, and regurgitation. Forty-tree specimens were classified as intestinal metaplasia and two as low-grade dysplasia.

### Tissue specimens and Immunohistochemistry (IHC) Staining

Endoscopic (mucosal) biopsy specimens from BE patients and matched tumor and adjacent normal specimens from EC patients, archived as formalin-fixed, paraffin-embedded material, were used for immunohistochemical analysis. Sections were routinely stained with hematoxylin and eosin and evaluated histopathologically. Tissue specimens were processed in a standard fashion, as described elsewhere 22. ENO1 was identified using an anti-ENO1 mouse monoclonal antibody at a concentration of 0.025 μg/ml (Abcam, Cambridge, UK). Immunostaining was with a peroxidase-based visualization DAKO LSAB^®^ kit, following the manufacturer's recommendations. Diaminobenzidine tetrahydrochloride was used as chromogen. Sections incubated with PBS instead of the anti-ENO1 antibody served as controls.

### Evaluation of staining

Histopathological examination was done by a senior pathologist (L.C.) who was unaware of the clinical findings. ENO1 expression into the cytoplasm and nucleus was empirically determined. IHC staining was graded using two semiquantitative measurements: staining intensity (0-4) and percentage of cells stained (0=no staining, 1=below 25%, 2=25%-50%, 3=50%-75%, and 4=75%-100%). A combined immunoreactive score (IRS) 23 was calculated as the product of staining intensity and percentage of stained cells.

### Determination of plasma ENO1 levels by ELISA

Peripheral blood samples were collected by aspiration in Vacutainer tubes containing 0.105 mol/liter sodium citrate, from EC patients (n=32) before surgery, BE patients (n=19), and healthy subjects (n=25). Blood was then centrifuged and plasma were aliquoted and stored at -80°C until use. ENO1 concentrations were determined using a specific ELISA kit (USCN Life Science Inc., Houston, TX 77082, USA). The lower detection limit of the assay was 39 pg/ml.

### Statistical analysis

Statistical analysis was performed using the GraphPad Prism 7 package (GraphPad Software, La Jolla, CA, USA). The Wilcoxon signed rank test and Mann-Whitney Rank Sum Test or Student's *t* test were used to evaluate statistically significant differences between datasets. The relationships between variables were investigated by the Spearman or Pearson correlation tests. One Way Analysis of Variance or Kruskal-Wallis One Way Analysis of Variance on Rank followed by All Pairwise Multiple Comparison Procedures (Dunn's Method) were applied to determine whether significant differences existed among the study groups. In determining the correlation between ENO1 tissue expression or plasma levels and prognosis, the median IRS or ENO1 concentration in the peripheral blood was taken as cut-off. Survival data were analyzed using the Kaplan-Meier method with a log-rank test for comparison. *P*<0.05 was considered to indicate a statistically significant difference.

## Results

### ENO1 expression was up-regulated in EC in comparison with adjacent non-cancerous tissue samples

*In situ* ENO1 protein expression status was analyzed by IHC in matched EC and adjacent normal tissue specimens (n=40). In tumor cells, positive expression was detected in 80% of EC patients, whereas in paired normal counterparts only 10% were positive. When patients were categorized by histological tumor subtype, ENO1 was expressed in tumor cells in 76.9% of ESCC cases and in 81.5% in EAC cases, and in matched normal tissues in 7.8% and 11.1%, respectively.

**Figure 1:**
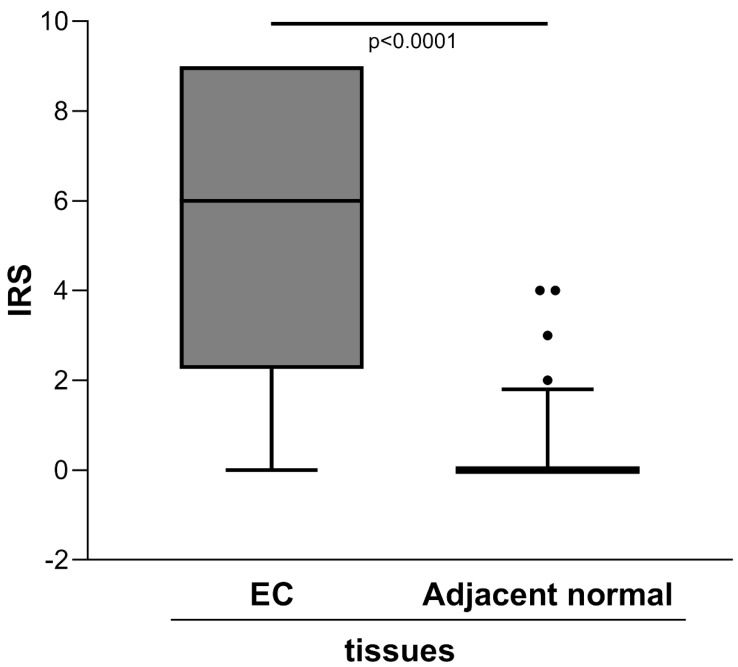
Quantitative immunohistochemical analysis of ENO1 protein expression in paired EC and normal adjacent tissues samples (n=40). The immunoreactive scores (IRS) were obtained as described in the Materials and Methods section. Median, 10^th^, 25^th^, 75^th^, and 90^th^ percentiles are presented as vertical boxes with error bars. Dots indicate outliers. *P*-values obtained by Wilcoxon signed rank test.

The semiquantitative assessment of staining (IRS) demonstrated markedly higher level of ENO1 in the clinical tumor samples of EC than in the adjacent normal tissues [IRS median (range): 6 (0-9) *vs.* 0 (0-4), *p*<0.0001] (Figure 1).

**Figure 2:**
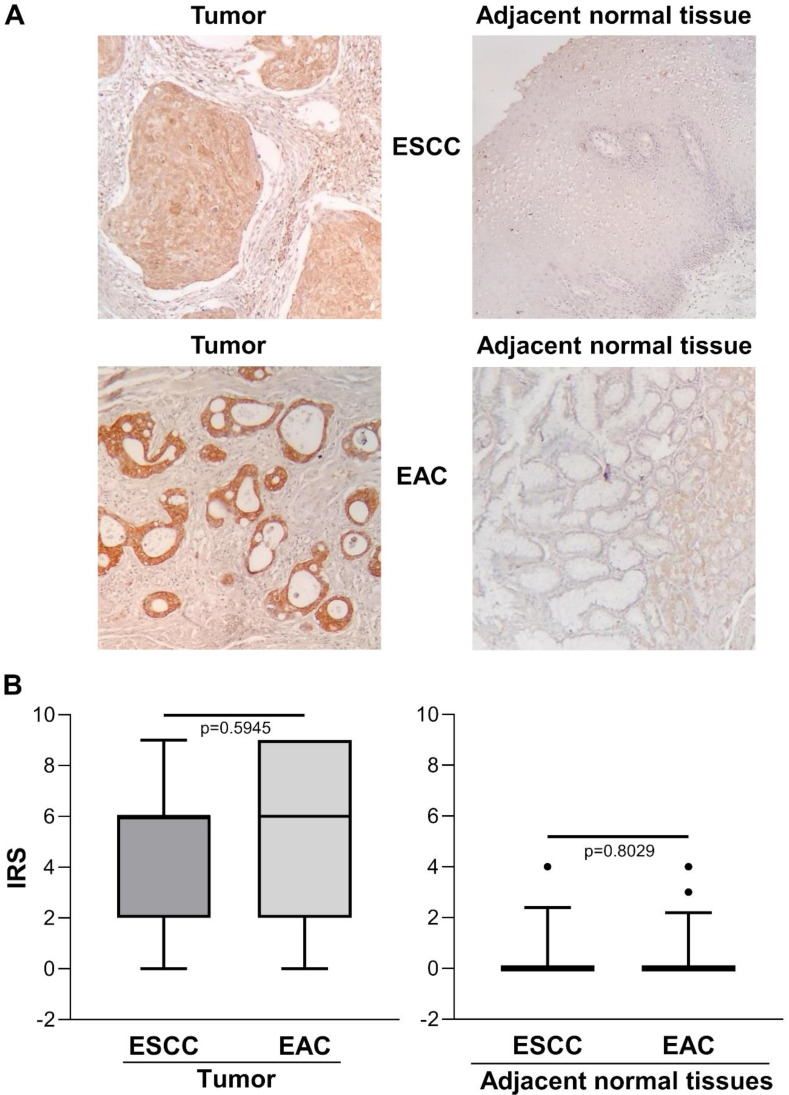
** (A)** Representative immunohistochemical analysis of ENO1 protein expression in ESCC and EAC tissues specimens and in paired normal adjacent esophagus mucosa sections (original magnification x100). No staining in negative controls (data not shown). **(B)** Quantitative analysis of immunostaining for ENO1 protein in ESCC, EAC and paired normal adjacent tissues samples (n=40). The immunoreactive scores (IRS) were obtained as described in the Materials and Methods section. Median, 10^th^, 25^th^, 75^th^, and 90^th^ percentiles are presented as vertical boxes with error bars. Dots indicate outliers. *P*-values obtained by Mann-Whitney Rank Sum Test.

Figure 2A shows representative images of immunohistochemical staining in ESCC (n=13), EAC (n=27) and the relative adjacent non-tumor tissue specimens. There was no significant difference between ESCC and EAC in either the tumor area or the adjacent normal tissue, with predominant strong IHC staining in the cytoplasm of basal cells and/or squamous epithelium [IRS median (range): ESCC=6 (0-9) *vs*. EAC=6 (0-9), *p*=0.5945 and IRS median (range): ESCC normal adjacent tissue=0 (0-4) *vs*. EAC normal adjacent tissue=0 (0-4), *p*=0.8029] (Figure 2B).

### ENO1 expression in BE versus EAC tissue samples

**Figure 3:**
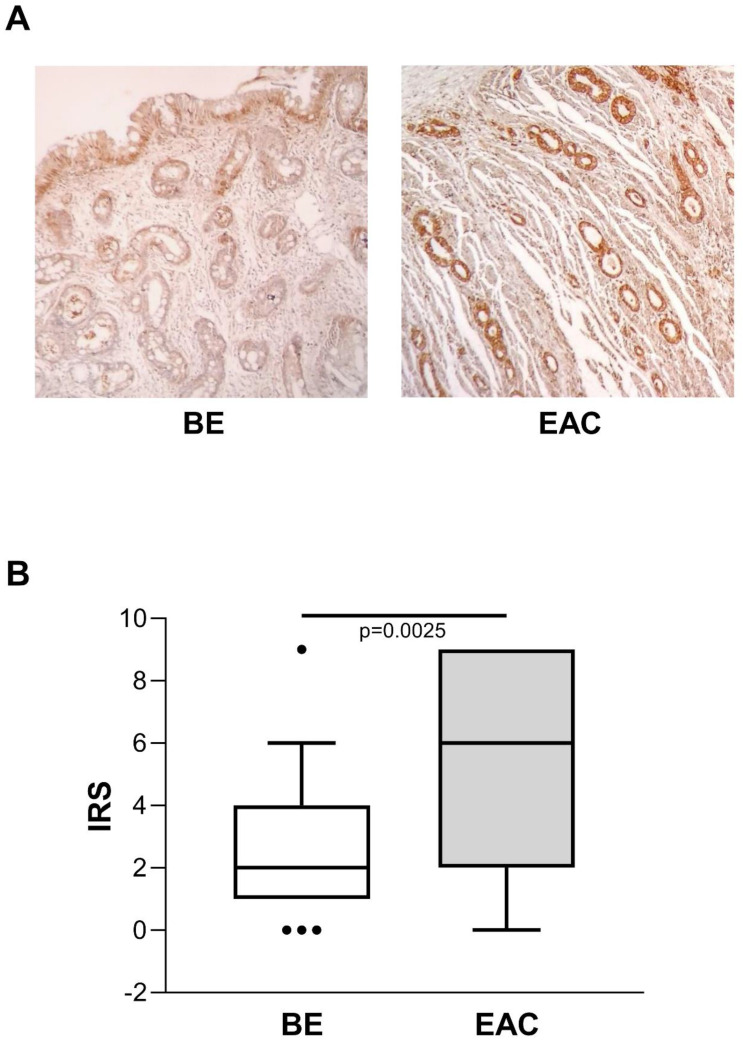
** (A)** Representative immunohistochemical analysis of ENO1 protein expression in BE and unrelated EAC tissues specimens (original magnification x100). No staining in negative controls (data not shown). **(B)** Quantitative analysis of immunostaining for ENO1 protein in BE and unrelated EAC tissue samples. The immunoreactive scores (IRS) were obtained as described in the Materials and Methods section. Median, 10^th^, 25^th^, 75^th^, and 90^th^ percentiles are presented as vertical boxes with error bars. Dots indicate outliers. *P*-values obtained by Mann-Whitney Rank Sum Test.

Considering the known risk of BE progression toward EAC, we also evaluated ENO1 protein expression in BE (n=45) and in unrelated EAC tissue samples by IHC, and compared the two. In BE cases, ENO1 expression was fairly homogeneous in squamous epithelium and in areas of intestinal metaplasia, with intense cytoplasmic staining in the specialized gastric fundus cells. Representative examples of staining in BE (n=45) and EAC (n=27) tissues are shown in Figure 3A. ENO1 IRS in BE tissues was lower than in unrelated EAC tissues [IRS median (range): BE=2 (0-9) *vs*. EAC=6 (0-9), *p*=0.0025] (Figure 3B).

### Correlation between tissue ENO1 expression and clinicopathological parameters in EC

Local ENO1 protein expression in EC was not associated with sex, age or tumor location (r=0.209, *p*=0.1942, r=0.259, *p*=0.1060, and r=-0.197, *p*=0.2210, respectively, Spearman correlation test). When EC cases were classified by degree of tumor differentiation, no statistically significant difference in ENO1 expression was found between grade 2 (n=14) (IRS mean±SE: 5.93±0.77), grade 2/3 (n=10) (IRS mean±SE: 3.84±1.21), and grade 3 tumors (n=16) (IRS mean±SE: 3.46±0.87) (One Way Analysis of Variance *p*=0.4671) (data not shown).

**Figure 4:**
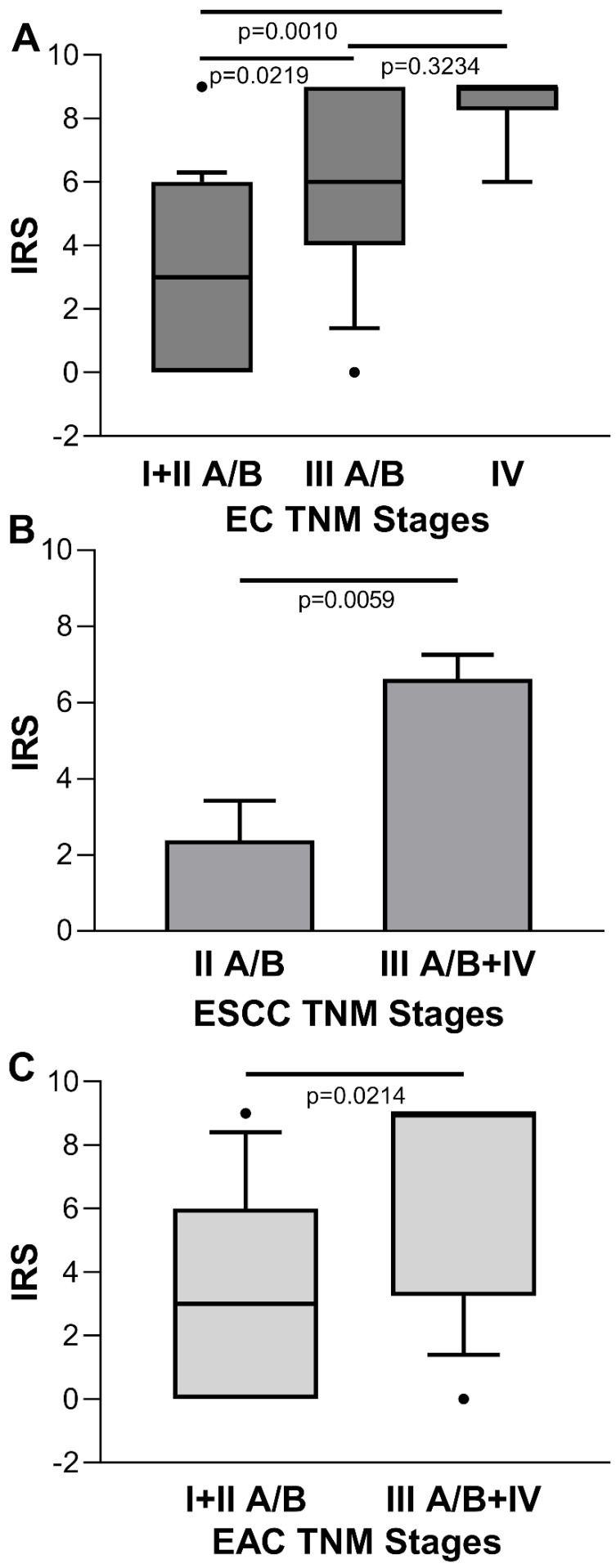
** (A)** Quantitative analysis of immunostaining for ENO1 protein in tissue samples from EC categorized by disease stage. Median, 10^th^, 25^th^, 75^th^, and 90^th^ percentiles are presented as vertical boxes with error bars. Dots indicate outliers. *P*-values obtained by Kruskal-Wallis One Way Analysis of Variance on Ranks followed by All Pairwise Multiple Comparison Procedures (Dunn's Method). **(B)** Quantitative analysis of immunostaining for ENO1 protein in tissue samples from ESCC categorized by disease stage. Results are expressed as mean±SE. *P*-values obtained by Student's *t* test. **(C)** Quantitative analysis of immunostaining for ENO1 protein in tissue samples from EAC categorized by disease stage. Median, 10^th^, 25^th^, 75^th^, and 90^th^ percentiles are presented as vertical boxes with error bars. Dots indicate outliers. *P*-values obtained by Mann-Whitney Rank Sum Test. The immunoreactive scores (IRS) were obtained as described in the Materials and Methods section.

Conversely, for the IRS of tumor cases stratified by disease stage, there was a statistical significant increasing trend towards the later stages [IRS median (range): stage I + II A/B=3 (0-9), stage III A/B=6 (0-9), stage IV=9 (6-9); *p*=0.0005 Kruskal-Wallis One Way Analysis of Variance on Rank; stage I + IIA/B *vs.* stage III A/B, *p*=0.0219; stage I + II A/B *vs.* stage IV, *p*=0.010; stage III A/B* vs.* stage IV, *p*=0.3234, Dunn's multiple comparisons test] (Figure 4A). No significant association was found between ENO1 expression quantified by IRS and EC stage in paired adjacent normal tissues (*p*=0.3813, Kruskal-Wallis One Way Analysis of Variance on Rank) (data not shown). When EC was classified according to the two major histologic types, differences in IRS values of the ESCC disease stage II A/B *vs.* III + IV, and EAC disease stage I + II A/B *vs.* III + IV were statistically significant [IRS mean±SE: 2.333±1.085* vs.* 6.571±0.685, *p*=0.0059, and IRS median (range): 3 (0-9) *vs.* 9 (0-9), *p*=0.0214, respectively] (Figure 4B and C).

### ENO1 was down-regulated in plasma of EAC patients

**Figure 5:**
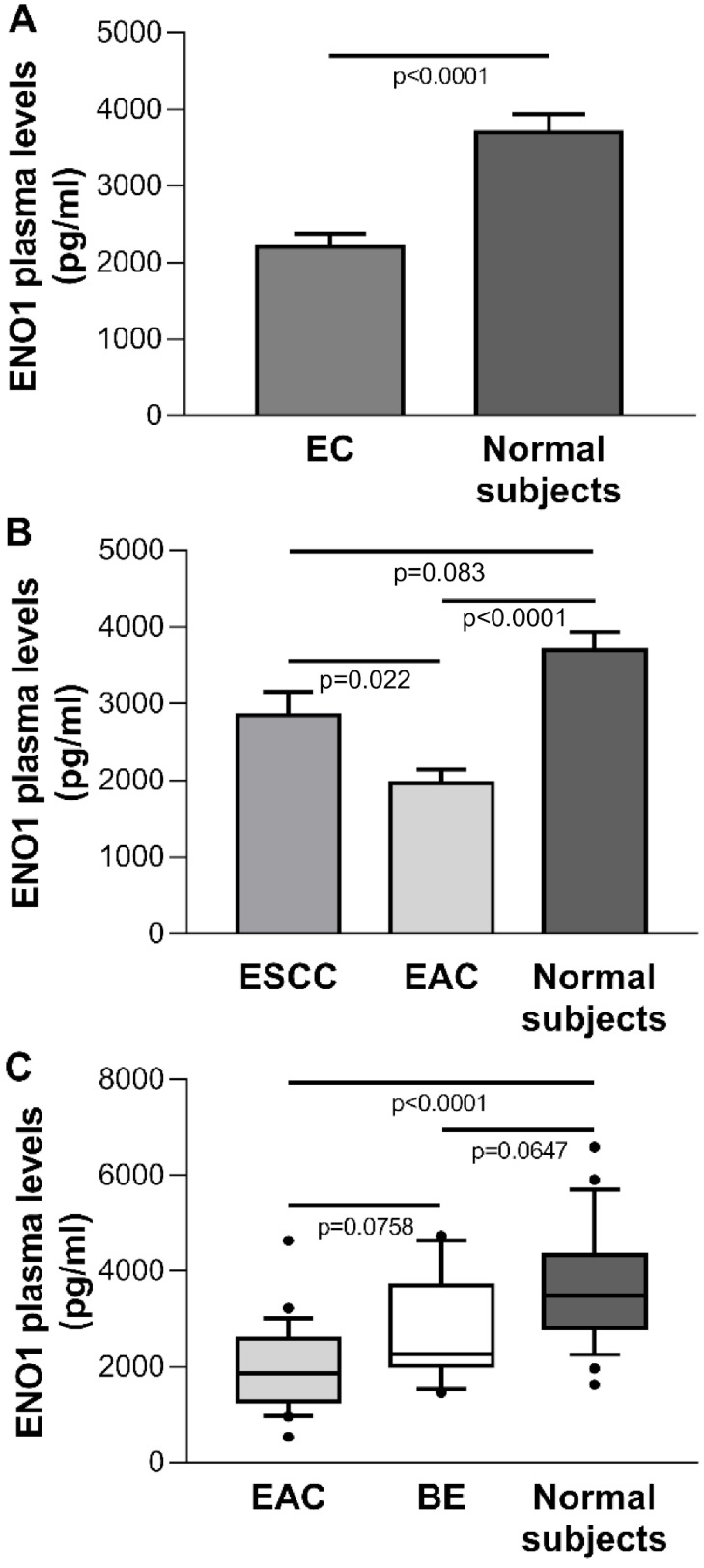
** (A)** Plasma ENO1 concentration by ELISA in EC patients and normal subjects. *P*-values obtained by Student's *t* test. **(B)** Plasma ENO1 concentration by ELISA in EC patients categorized by histological type of tumor (ESCC and EAC) and normal subjects. Results are expressed as mean±SE. ENO1 concentration (pg/ml).* P*-values obtained by Unpaired Studentì's *t*-test. **(C)** Plasma ENO1 concentration by ELISA in normal subjects, BE and EAC patients. Median, 10^th^, 25^th^, 75^th^, and 90^th^ percentiles are presented as vertical boxes with error bars. Dots indicate outliers. *P*-values obtained by Kruskal-Wallis One Way Analysis of Variance on Ranks followed by All Pairwise Multiple Comparison Procedures (Dunn's Method).

The mean±SE ENO1 level in plasma of the EC group (n=32) was 2183±171 pg/ml, which was significantly below the concentration found in healthy donors (n=25) (3693±246 pg/ml, *p*<0.0001) (Figure 5A). When EC cases were classified by histological type, in ESCC patients (n=8) circulating ENO1 concentrations did not differ from those in normal subjects (mean±SE pg/ml: 2849±309 *vs* 3693±246, *p*=0.083), but were significantly higher than in EAC patients (n=24) (mean±SE pg/ml: 2849±309 *vs* 1961±185, *p*=0.022). By contrast, in the EAC group plasma ENO1 levels were significantly lower than those in healthy donors (mean±SE pg/ml: 1961±185 *vs* 3693±246, *p*<0.0001).

### ENO1 plasma levels in BE and EAC patients

To determine whether circulating ENO1 may be an important risk factor for progression from BE to EAC, the plasma concentrations of this enzyme in normal subjects, BE cases (n=19), and EAC patients were compared. A trend toward increased ENO1 levels was found in the sequence EAC patients, BE patients, healthy subjects [median (range) pg/ml: 1867 (538-4636), 2273 (1461-4737), 3494 (1629-6596), respectively, *p*<0.0001, Kruskal-Wallis One Way Analysis of Variance on Ranks; EAC *vs.* BE, *p*=0.0758; EAC *vs*. normal donors, *p*<0.0001; BE* vs.* normal donors, *p*=0.0647 (Dunn's multiple comparisons test)] (Figure 5C).

### Correlation between circulating ENO1 level and clinicopathological parameters in EC

ENO1 plasma levels in EC was not associated with age, sex, or tumor location (r=-0.1093, p=0.5515, Pearson correlation test; r=-0.1393, p=0.4472, and r=0.2158, p=0.235, Spearman correlation test, respectively). When EC cases were classified by degree of tumor differentiation, no statistically-significant difference in circulating ENO1 levels was found between grade 2 (n=11), grade 2/3 (n=7), and grade 3 tumors (n=14) (mean±SE pg/ml, 2143±178, 2161±384, 2225±326, respectively; *p*=0. 9773, One Way Analysis of Variance), even when EC cases were categorized by histological type (data not shown).

Further, when plasma levels of EC cases were stratified by disease stage, there was no statistically significant difference [mean±SE pg/ml: 2346±251, stage I + II/B (n=15); 2266±306, stage III A/B (n=11); 1624±324, stage IV (n=6); *p*=0.2940, One Way Analysis of Variance], even when EC cases were categorized by histological type (data not shown).

### Overall survival analysis

To define the clinical significance of local and circulating ENO1 protein levels in EC, the correlation between ENO1 IRS and plasma concentrations and overall survival was analyzed. The Kaplan-Meier curves for overall survival of all EC patients showed that neither parameter was significantly associated with survival (ENO1 IRS and plasma concentration: *p*=0.7258 and *p*=0.8501, respectively), even when EC cases were subdivided by histological type (ESCC ENO1 IRS and plasma concentration: *p*=0.1125 and *p*=0.5835, respectively; EAC ENO1 IRS and plasma concentration: *p*=0.3739 and *p*=0.5328, respectively).

## Discussion

ENO1, as a key glycolytic enzyme, may play pivotal role in aerobic glycolysis (the so-called Warburg effect) contributing to tumor progression of numerous cancers.

The present study targeted the hitherto unexamined expression of ENO1 protein, using an immunohistochemical method, in tumor and matched adjacent normal tissue sections from EC patients, as well as ENO1 circulating levels; their clinical relevance was also examined. It showed that: i) ENO1 production is abnormally elevated in cancer cells in both ESCC and EAC, increasing during tumor stage progression and in non-dysplastic BE, the premalignant condition that predisposes to the development of EAC, ii), unexpectedly, EAC patients exhibited significantly lower ENO1 plasma levels compared to normal subjects, and iii) both *in situ* and systemic ENO1 levels showed no significant correlation with overall survival of the patients. These results suggest that upregulation of ENO1 may accelerate the cells' glucose metabolism in esophagus cells as early event in the Barrett's adenocarcinoma transition and in association with tumor progression.

In agreement with numerous studies on different human cancer types 13-19, 24-30, the study found that ENO1 protein was up-regulated in EC tissues in comparison with adjacent non-tumorous tissues.

In addition to glycolytic activity, ENO1 appears to have various cellular functions and subcellular localizations, performing important role in other pathophysiological processes in cancer 31. When located in the nucleus, the enzyme may inhibit transcription of the proto-oncogene c-myc, acting as a transcriptional repressor 32. Moreover, ENO1 expressed on the cell surface can function as a plasminogen receptor, and contributes to cell invasion and metastasis 33. In the cytoplasm, ENO1 provides a rapidly available supply of ATP to cells and, interacting with the cytoskeletal system, supports the rapid growth, proliferation, and movement of cancer cells 20. ENO1 also strengthens the infiltration ability of monocytes and macrophages, and it can participate in tumor formation by controlling the expression of the c-myc oncoprotein through the Notch signaling pathway 34.

IHC analysis failed to detect enhanced ENO1 membrane or nuclear localization in EC tissue specimens, indicating that the enzyme activity is likely involved in regulating glycolysis rather than in controlling transcription and/or extracellular matrix remodeling.

EC includes two main subtypes: ESCC, which develops from squamous epithelium undergoing inflammatory, hyperplastic, and dysplastic changes, and EAC, which arises through metaplastic intestinal-type changes replacing the squamous epithelium. The study found that ENO1 *in situ* protein levels did not differ significantly between ESCC and EAC patients, suggesting that ENO1 expression is not associated with the pathological tumor type. Moreover, statistical analysis revealed that high ENO1 immunohistochemical expression levels in both ESCC and EAC were significantly associated with disease stage, but not with other clinical features such as age, gender, tumor location, or degree of tumor differentiation. A positive correlation has been reported between elevated *in situ* ENO1 protein expression and cancer progression in pancreatic and hepatocellular carcinoma patients 25, 28. By contrast, in lung, colon, and nasopharyngeal cancer tissues, ENO1 expression appears inversely correlated with disease stage 29, 35. These results suggest that ENO1 may play different roles in tumor growth, depending on the type of cancer, although specific action mechanisms remain unclear.

BE is considered a complication of chronic gastroesophageal reflux, and represents a major risk factor for development of EAC. Currently, alongside dysplasia, few molecular markers may be used to delineate a population of BE patients at high risk for cancer 36, 37. In the present study, ENO1 protein was found to be *in situ* overexpressed already in non-dysplastic BE, as well as in EAC compared to normal surrounding mucosa, with a progressive increase along the sequence normal, premalignant and neoplastic epithelium. ENO1 might thus be a useful biomarker of development of BE in EAC, although further studies will be needed to fully document the diagnostic and prognostic value of ENO1 in this process.

Although ENO1 has been extensively evaluated using proteomics and IHC in tumor cell lines and tissues, few studies have examined circulating levels of the enzyme in cancer patients. ENO1 may be delivered into bloodstream by tumor cell necrosis and turnover or unconventional secretory pathways. Abnormally higher circulating ENO1 levels were reported in non-small-cell lung cancer (NSCLC) 38 and pancreatic cancer patients 25. Unexpectedly, we found that EAC, but not ESSC patients had in plasma significantly lower ENO1 level compared with healthy individuals. Interestingly, circulating ENO1 levels decreased progressively in normal, precancerous condition of the esophagus and EAC sequence, exactly in the opposite way to the tissue expression of the protein. Therefore, also in the periphery, BE mucosal abnormality, a clearly recognized risk factor for EAC development, is associated with altered plasmatic ENO1 levels that may predate the clinical diagnosis of malignancy.

It is tempting to speculate that the increase in glucose uptake and elevated proliferation rates associated with the *in situ* tumor growth may provoke a rapid consumption of ENO1, the master regulator of tumor metabolism, and in turn an aberrant release of the enzyme*.* Otherwise, circulating ENO1 can be degraded by some activated proteases secreted from tumor or released from tumor cell death 39*.* Moreover, it is well documented that ENO1 might act as an autoantigen in several cancer 40, 41, reflecting the greater immunologic reactivity and enhanced immune surveillance for cancer cells.

ESSC and EAC are clearly distinct cancers with differences in risk factors, histopathology and molecular profile. Tumor microenvironment could potentially drive this divergence. Since the tissue expression of the protein ENO1 did not differ significantly in the two EC subtypes, a potential mechanism accounting for decreased levels of circulating ENO1 in EAC patients could be a more prompt clearance of the immunocomplexes formed by ENO1/anti-ENO1 antibodies, released into the circulation during tumor growth. However, taking into account the discrepancies between our results and those of other studies, the clinical significance of circulating ENO1 in EC requires further exploration, including the quantification of the production of ENO1 autoantibodies.

Previous investigations report an association between *in situ* ENO1 overexpression and a worse clinical outcome in a variety of tumors, such as pancreatic adenocarcinoma 25, hepatocellular carcinoma 28, 42*,* glioma 14, breast cancer 15, and head and neck cancer 17. Conversely, most NSCLC patients, who show down-regulated ENO1 expression, exhibit a poor prognosis 16. It emerged from the present study that, in EC, local ENO1 protein upregulation was related only to tumor progression and not to overall survival. The different findings reported by other studies and the present work may be related to the types of cells investigated, and might also be closely related to ENO1 expression level.

As reported above, besides its innate glycolytic function, ENO1 may play other roles in tumorigenesis depending on its cellular localization. This study found that ENO1 in EC cells was mainly localized in the cytoplasm, consistent with its supporting role in the high metabolic rate of tumor cells. As occurs in breast cancer and NSCLC 15, 16, 43, ENO1 surface and nuclear forms may be involved in cancer invasion and metastasis, as well as in transcriptional repression, apparently inhibiting cell growth and accelerating apoptosis and necrosis 44. Moreover, in gastric cancer, ENO1 protein levels are associated with chemoresistance 45. Thus, the various cellular localizations, as well as the different pathways regulating ENO1 functions in the different types of tumor cells, may account for the inconsistence of the experimental results reported thus far.

In conclusion, alongside this explorative study, future in-depth investigations are needed to further elucidate the specific clinical value of ENO1 in EC, in consideration of its possible utility in delineating a population of patients with BE at high risk of cancer, and representing a new therapeutic target to counteract the growth and progression of this aggressive tumor.

## Figures and Tables

**Table 1:** Clinical, demographic and pathological features of the study population

Characteristic	Number
**Age**	
Years, median (range)	68 (44-83)
**Gender**	
Female/Male	10/30
**Histopathology**	
Adenocarcinoma	25
Barrett's-associated adenocarcinoma	2
Squamous cell carcinoma	13
**Histologic grade (G)**	
Well differentiated (G1)	0
Moderately differentiated (G2)	14
Moderately/poorly differentiated (G2/G3)	10
Poorly differentiated (G3)	16
Undifferentiated (G4)	0
**Tumor location**	
Hypopharynx- esophagus junction	2
Cervical esophagus	2
Upper thoracic esophagus	1
Medium/Lower esophagus	12
Cardia**s**	23
**Tumor**	
*In situ*	0
T1	1
T2	15
T3	19
T4	5
**Lymph node metastasis**	
Absent (N-)	10
Present (N+)	30
**Metastasis**	
Absent	38
Present	2
**Staging**	
I	1
II	16
III	17
IV	6

## Abbreviations

BE: Barrett's esophagus
EAC: esophageal adenocarcinoma
EC: esophageal cancer
ENO: Enolase
ESCC: esophageal squamous cell carcinoma
IHC: immunohistochemistry
IRS: immunoreactive score
NSCLC: non-small-cell lung cancer
TNM: tumor node metastasis
